# Temporary Albuminuria

**Published:** 1853-03

**Authors:** 


*Temporary Albuminuria.* By Dr. BEGBIE.—Dr. J. W. Begbie alludes to the phenomenon of albuminuria in the following diseases:

1. *Scarlatina Simplex.*—He confirms his former statement that about the period of desquamation, albumen can *almost* always be found; its presence is associated with renal epithelium, but not with casts of tubes.
2. *Cholera.*
3. *Erysipelas.*—Usually at resolution or during convalescence. Its presence is not constant, nor its quantity great.

The albuminuria in these three cases is called *desquamative.*

4. *Scarlatinal Dropsy.*—The albuminuria may, or may not, be temporary—blood exudation-corpuscles, and casts of tubes, accompany it.

The albuminuria in this case is termed *inflammatory.*

5. *Pneumonia,* at the period of resolution, in almost all cases.
6. *Typhus* and *Typhus Abdominalis* (typhoid.)—From a consideration of the period when the albumen is observed in these last-named diseases, Dr. Begbie terms it *critical albuminuria.*—*London Monthly Journal,* Oct. 1852.

